# Evaluating a Comprehensive Multimodal Outpatient Rehabilitation Program to Improve the Functioning of Persons Suffering from Post-acute Sequelae of SARS-CoV-2 infection (PASC): A Randomized Controlled Trial

**DOI:** 10.21203/rs.3.rs-7375832/v1

**Published:** 2025-08-18

**Authors:** Benjamin Abramoff, Timothy Dillingham, Ava-Kathleen Rybicki, Frances Shofer, Emily McGinley, Shefali S. Verma, Monica Coran Kuns, John T. Barry, Liliana E. Pezzin

**Affiliations:** Department of Physical Medicine and Rehabilitation, University of Pennsylvania Perelman School of Medicine, Philadelphia PA; Department of Physical Medicine and Rehabilitation, University of Pennsylvania Perelman School of Medicine, Philadelphia PA; Department of Physical Medicine and Rehabilitation, University of Pennsylvania Perelman School of Medicine, Philadelphia PA; Department of Emergency Medicine, University of Pennsylvania Perelman School of Medicine, Philadelphia PA; Institute for Health and Equity, Medical College of Wisconsin, Milwaukee WI; Pathology and Laboratory Medicine, University of Pennsylvania Perelman School of Medicine, Philadelphia PA; Good Shepherd Penn Partners, Philadelphia PA; Good Shepherd Penn Partners, Philadelphia PA; Institute for Health and Equity, Medical College of Wisconsin, Milwaukee WI

**Keywords:** COVID-19, post-acute sequela, long COVID, rehabilitation, outcomes, comparative effectiveness research

## Abstract

**Background:**

About 10–20% of persons who contract SARS CoV-2 will experience persistent post-acute sequelae of SARSCoV-2 infection (referred here as PASC). Given that persistent symptoms are heterogeneous with multisystem involvement, recent consensus recommendations suggest that a holistic rehabilitation program may be required to manage PASC and restore function. While treatments offered at emerging outpatient COVID recovery clinics are being informed by previous similar diseases, the need is great for a better understanding of the unique needs of this growing population and for tested, efficacious rehabilitation programs to address them.

**Methods/Design:**

Data from a large and diverse ongoing longitudinal survey of persons positive for COVID-19 at the study health system will serve as the sampling frame from which to enroll PASC study patients. The targeted six-week program will comprise a core set of therapies including individually titrated isometrics, strengthening of accessory breathing muscles and diaphragm, resistance and aerobic conditioning, and neuropsychological and cognitive remediation tailored to patients’ needs. Using a randomized controlled trial design, the effectiveness of the supervised multimodal rehabilitation intervention will be compared to that of a self-guided (active control) group receiving patient education materials adapted from the World Health Organization guidelines for PASC management. In addition to walking speed, a widely used global measure of aerobic capacity and endurance, and patient-reported health and functioning (primary outcomes), we will assess intervention effectiveness on: cognitive functioning, pain, fatigue, tension, stress, anxiety, and depression, and self-management of PASC symptoms (secondary outcomes). Outcomes will be measured at 8 weeks and at 90 day’s post- study entry to examine sustainability of effects. Recruitment is ongoing.

**Discussion:**

It is unlikely that COVID-19 vaccines will lead to the end of PASC syndrome given the emergence of increasingly infectious variants and the high numbers of individuals with continued symptoms several months from initial infection. High rates of vaccine hesitancy and refusal will also contribute to the persistence of both COVID-19 and PASC. Given the dearth of rigorous scientific evidence regarding effective assessment and treatment of PASC and unresolved questions concerning post-COVID rehabilitation care, the results of this study will have significant implications for both policy and program development.

**Trial registration:**

ClinicalTrials.gov ID: NCT06156202

## BACKGROUND

There is overwhelming evidence that for many individuals, the effects of COVID-19 does not end after the resolution of the acute viral infection.^1–10^ In fact, studies suggest that millions of individuals in the United States and worldwide continue to have persistent symptoms following the resolution of their acute viral illness. Conservative estimates suggest that roughly 6.2% of individuals have persistent symptoms^11^ three months after acute infection while other prospective studies show up to 79% of hospitalized patients continuing to experience symptoms at 3 months.^12^ These symptoms are seen among the full spectrum of patients, from those managed at home to those hospitalized with severe illness.^12–13^ Persistent symptoms are not short-lived with evidence of continued sequalae lasting more than 3 years following acute infection.^14^ These long-term symptoms affect all age groups,^13^ genders,^9^ races, and are seen throughout the world.^10^

The effects of PASC are widespread and have been reported to affect nearly every organ system in the body.^17–18^ Some of the most commonly reported symptoms include fatigue (mean 58%; CI 42–73%), cognitive changes (attention, mean 27%; CI 19–36% and memory, mean 16%; CI 0–55%), and dyspnea (mean 24%, CI 14–36%).^18^ Other common symptoms reported include weakness, chest pain, gastrointestinal issues, loss of taste and smell, skin changes, neuropsychological issues, and insomnia.^15–16^ These changes have been noted to lead to significant occupational, financial and social distress.^14,17^ Patients have been noted to be unable to return to work and normal activities which can also lead to significant financial impacts.^7^

Given the widespread, devastating effects of PASC, it is imperative that effective treatments be identified to alleviate symptoms and restore previous function.^18–26^ There is emerging evidence about the potential benefits of rehabilitation programs to improve physical function and symptoms in individuals with Long COVID. Despite this, there remains a lack of high quality randomized clinical trials investigating rehabilitation interventions. Previous studies were limited by underrepresentation of female participants, lack of allocation concealment, lack of blinding, and other forms of bias. Previous studies also have also looked at limited outcome domains with fatigue particularly being under-investigated as an outcome despite being the most common sequelae of Long COVID.^27–28^ Additionally, there remains a high level of uncertainty regarding adverse events^29^ with a need for clinical trials with close monitoring of adverse events. Furthermore, many studies do not clearly describe the specific rehabilitation techniques that are needed to guide clinical care and fidelity to rehabilitation interventions.

Given that persistent symptoms are heterogeneous with multisystem involvement, recent consensus recommendations suggest that a holistic rehabilitation program may be required to manage PASC and restore function. While treatments offered at emerging outpatient COVID recovery clinics are being informed by previous similar diseases, the need is great for a better understanding of the unique needs of this growing population and for tested, efficacious rehabilitation programs to address them.

### Study Aims

To address these important gaps, we are developing and evaluating the effectiveness of a patientcentered, interdisciplinary, multimodal comprehensive rehabilitation program among patients with post-acute sequelae of SARS-CoV-2 infection (PASC). The targeted six-week program will be comprised of a core set of therapies, including individually titrated stretching and flexibility, strengthening of accessory breathing muscles and diaphragm, resistance and aerobic conditioning, and vestibular rehabilitation, supplemented by neuropsychological and cognitive remediation tailored to patients’ needs. Using a randomized controlled trial (RCT) design, the effectiveness of the supervised intervention will be compared to that of a self-guided (active control) group receiving a one-time in-person assessment and patient education materials adapted from the WHO guidelines.^30^ In addition to walking speed, a widely used global measure of aerobic capacity and endurance, and patient-reported health and functioning (primary outcomes), we will assess the intervention effectiveness on: (1) cognition, (2) pain, (3) fatigue, (4) anxiety, and depression, and (5) self-management (secondary outcomes). Outcomes will be measured at fixed points in time at 8 weeks (shortly after therapy completion) and at 90 day’s post-study entry to examine sustainability of effects.

If found to be effective, the intervention has the potential to substantially reduce (if not eliminate) disabling persistent symptoms, promote return to pre-infection major usual activities, and improve the quality of life of persons suffering from PASC. In addition, because the therapy strategies are well defined and documented, if found effective, the intervention may be readily implemented at other centers.

## METHODS/DESIGN

### Overview of Study Design

Central to this study are efforts to develop and test a patient-centered rehabilitation intervention to improve the functioning and quality of life of patients experiencing PASC. Using the Penn Medicine Bio Bank (PMBB), an institutional resource whereby consented patients provide sociodemographic, COVID-specific, and other clinical information, as a sampling frame, we will employ a randomized design to evaluate the effectiveness and cost-effectiveness of a supervised interdisciplinary, multimodal outpatient rehabilitation intervention intended to improve physical, cognitive, psychological, and social functioning, thereby improving the health-related quality of life of persons experiencing persistent post-SARS-CoV-2 infection sequelae. The intervention will be assessed relative to an active, self-guided rehabilitation program control group receiving one- time in-person assessment and the WHO Guide “Support for rehabilitation Self-Management after COVID-19 Related Illness.”

The analysis will estimate the impact of the intervention on patient outcomes and on costs. Measures of these dependent variables will be derived from a combination of primary and secondary data. Regression models will be used to control for chance differences among participants in the two randomized groups with respect to characteristics that might influence outcomes. Estimates of treatment-control group differences generated by these models will determine the extent to which observed differences in clinical (e.g., walking speed) and patient-reported (e.g., SF-36) outcomes are attributable to the comprehensive rehabilitation program intervention.

### Research Setting

The study protocol is offered at three sites in the greater Philadelphia area, which have been chosen for being in geographically and socioeconomically diverse areas: the Penn COVID Recovery Clinic in central Philadelphia, the COVID clinic at Radnor, a suburb west of Philadelphia in PA, and at Cherry Hill, NJ. All sites are staffed by University of Pennsylvania affiliated providers who have been trained on study protocol.

### Data Sources

The PMBB database is a large inception cohort recruiting patients and workers from the University of Pennsylvania Health System. Since its inception in 2013, recruitment and consenting has been conducted by a core group of trained clinical research coordinators (CRCs) working specifically for the biobank. As of April 2020, and up to June 2021, CRCs were deployed to various high-volume testing locations throughout the health system to enroll COVID participants. At that time, enrollment was also extended to community individuals living in the greater Philadelphia region. Respondents across the adult age span (> 18 years old) and of all races, ethnicities, and sexes were invited to participate.

Leveraging this infrastructure, we developed a protocol for longitudinal data collection of COVID+ (and a subset of COVID-) individuals at 3-month intervals to document persistence (or resolution) of symptoms and development of new symptoms over a 12-month period, studying both newly- and previously infected individuals. Implementation of our IRB-approved follow-up surveys follows the PMBB protocol, including a RedCap-assisted data collection instrument, use of trained CRCs, data monitoring and quality control, and initial data processing. The first follow-up, targeting persons who enrolled in the PMBB since August 2020, began fielding February 2021. More than 17,000 such individuals were enrolled as of December 2022. These individuals comprise the sampling frame from which we identify the subset of COVID + individuals with symptoms persisting > 12 weeks since diagnosis.

In addition to the PMBB, our study uses two additional data sources: structured in-person patient assessments, conducted at baseline and at 8-weeks, and online assessments conducted 90-days poststudy entry. The study protocol has been approved by the University of Pennsylvania (UPenn) and Medical College of Wisconsin (MCW) Institutional Review Boards.

### Patient Population

The study targets adult (18 or older) individuals with a confirmed diagnosis of COVID-19 who report symptoms persisting for more than 12 weeks in at least two domains: fatigue/endurance and cognitive functioning/memory. Identification is based on responses to specific COVID symptomatology and associated impairments ascertained during the longitudinal PMBB COVID surveys and confirmed during the phone screener interview. Subjects must score a 2 or greater on the fatigue assessment scale (maximum of 12 points) and score 10 or greater on the MoCA Blind memory/thinking scale (maximum of 22 points).

Exclusion criteria consist of patients participating in a clinical trial, those with a history of severe dementia or Alzheimer’s disease, history of chronic fatigue syndrome, those physically or medically unable to participate in our structured rehabilitation program as well as non-English, non-Spanish speaking individuals.

### Study Procedures

#### Recruitment.

PMBB respondents are identified on a rolling-basis. Once potentially eligible individuals have been identified from the follow-up PMBB surveys, the clinical research coordinator contacts them by phone to describe the study, obtain consent, and conduct a brief structured screener to confirm eligibility. Subjects providing verbal consent to participate have an in- person baseline assessment scheduled at their earliest convenience.

#### Baseline Visit.

After obtaining written informed consent, the clinical research coordinator conducts the performance-based assessments and administers a computer-assisted RedCap interview of validated patient-reported instruments.

#### Randomization.

Randomization occurs after completion of the baseline assessment. At that point, a computerized algorithm assigns individuals randomly to one of the study’s two arms at a ratio of 2 (supervised rehabilitation group): 1(self-guided, active control group).

#### Follow-up visits.

The Clinical Research Coordinator meets with the participants at approximately 6 to 8 weeks from study entry to collect performance and self-report data. Visits are scheduled to coincide with regularly scheduled appointments at the patient’s clinic site for individuals in the supervised therapy group and at the clinic where baseline assessment was conducted for individuals in the self-guided, active control group.

#### Compensation.

Participants will receive $50 at the time of initial assessment and $75 at the 6-week and 90-day follow-up assessments as reimbursement for their time, for a total payment of $200.

#### Data and Safety Monitoring Plan.

Adverse events are reported to the appropriate IRBs. To ensure the safety of participants and the validity and integrity of the data, a data and safety monitoring board (DSMB) has been established and meets approximately every six months. The DSMB includes senior investigators with expertise in PASC rehabilitation and biostatistics.

### Study Interventions

Supervised Multimodal Rehabilitation Program. Following the guidelines proposed by the national panel of experts led by Dr. Abramoff and recently published in Consensus Statement on PASC,^31–34^ the exercise program will be targeted to the person’s physical capacity with the goal of slowly advancing the exercise duration or intensity to effect physiological strengthening and increased physical function.

Progression through the supervised rehabilitation program is personalized and designed based on performance during intake tests and previous sessions. Subjects undergo 12 one-hour sessions over the course of six-weeks, two sessions per week. In certain circumstances, the 6 weeks of therapy may be extended to 8 weeks to accommodate unforeseen issues— unrelated to the study— that can disrupt continuous enrollment for subjects.

Subjects begin with structured breathing/respiratory exercises which will include diaphragmatic breathing, pursed lip breathing, pranayama breathing with intervals based on performance with spirometry, paced breathing, and breath control. Following the initial visit, if the subject denies having any respiratory complaints and can tolerate submaximal aerobic activity, breathing/respiratory exercises are omitted. All subjects receive an individually titrated symptom-guided physical therapy program to improve physical functioning. Subjects are also educated on recognizing perceived exertion and will be monitored by heart rate and the Borg Rating of Perceived Exertion Scale (BRPE).^35^ Activity is then slowly advanced as tolerated as long as it does not cause worsening of symptoms either during the session or in the evening and days after the session. If the symptoms worsen, the subject are returned to the previously tolerated level of exertion.

Subjects presenting with severe symptoms or impairments in physical functioning begin with upper and lower extremity light muscle strengthening at sub-maximal levels. Once they are able to tolerate, subjects move onto the aerobic program as discussed in the next paragraph. If unable to tolerate, physical therapy sessions will focus on breathing exercises and passive stretching. Subjects who have mild-moderate symptoms or impairments in physical functioning will begin with light aerobic exercises at sub-maximal levels (BRPE 12–14, 50–70% of maximum predicted heart rate for age). If patients do not have any exacerbation of their symptoms, each week the intensity or duration of the exercise will be advanced to provide a strengthening and conditioning effect while maintaining the subject at sub-maximal BRPE.

All subjects will receive cognitive therapy as well. This program will consist of both compensatory and restorative approaches. Compensatory strategies will be selected based on performance during intake tests and participant selected areas of concern, such as attention, memory, word finding, information processing, and executive functioning. Personalized compensatory strategies will be developed and trained to support impacted areas, such as environmental modifications and access to external memory aids (i.e. using a smartphone). Meta-cognitive skills training will be incorporated to teach subjects about self-monitoring and improving self-awareness by identifying strategies that work or don’t work, and why. Restorative cognitive therapy will provide the subject with cognitive exercises to improve areas of impairment. Depending on the area of impairment, restorative treatments to be offered include attention process training/functional hierarchical attention tasks, use of internal memory strategies, functional executive functioning exercise (e.g. Time Pressure Management), auditory processing exposure, and attentive reading and constrained summarization for thought organization/word finding. Finally, subjects will be educated on healthy diet/hydration, pacing and energy conservation, sleep/sleep hygiene and identification/avoidance of symptom triggers. Self-rating of cognitive effort and activity tracking will be incorporated to further promote awareness development for cognitive fatigue to reduce over-exertion.

Self-Guided Active Control Group. Subjects randomized to the self-guided program group receive the results of their in-person baseline assessment as well as the WHO Guide “Support for rehabilitation Self-Management after COVID-19 Related Illness.”^36^ This guide consists of ways to manage breathlessness, difficulty with voice, eating, drinking and swallowing; problems with attention, memory and thinking clearly; limitations in activities of daily living, manage stress and mood dysfunction and advice for when to contact healthcare professionals.

### Intervention Fidelity

Our efforts to maximize the quality and consistency of the intervention include careful training of physical and speech/cognitive therapists and monitoring of adherence to the intervention to evaluate consistency across study sites. The therapists delivering the supervised rehabilitation intervention are experienced practitioners, well-versed in principles and strategies needed for effective rehabilitation of chronic disease patients. To provide data on intervention content for secondary analyses, the therapists complete a checklist following each session to indicate the activities covered and any deviations from the program.

### Data Collection and Management

This study has a research core comprised of an experienced clinical research coordinator, a senior biostatistician, a senior econometrician, and a programmer/data analyst who are jointly responsible for the: 1) development of the computer-assisted data collection system, 2) staff training and certification in data collection, 3) randomization procedures, 4) data monitoring and quality control, 5) data processing, and 6) data analysis. The group also prepares regular reports for internal and external monitoring of progress toward study milestones and provides blinded and unblinded data requests for DSMB meetings.

### Study Measures

Assessment instruments will rely on readily available, validated measures. Consistent with the multidisciplinary PASC Consensus Guideline Statement, the 6-minute walk test (**6MWT)** will be our primary **performance-based** clinical outcome.^37^ The 6MWT is a widely used global measure of aerobic capacity and endurance. It is a simple and practical test that measures self-selected walking speed that has been endorsed by the Society of

Critical Care Medicine to evaluate physical function after critical illness.^38^ The Medical Outcomes Study Short Form Health Survey, **MOS-SF-36**,^39^ an assessment of general health and functioning, will be our primary **patient-reported** outcome. Designed for use in clinical practice and research, the SF-36 includes one multi-item scale that assesses eight health concepts: limitations in physical activities because of health problems; limitations in social activities because of physical or emotional problems; limitations in usual role activities because of physical health problems; bodily pain; general mental health (psychological distress and well-being); limitations in usual role activities because of emotional problems; vitality (energy and fatigue); and general health perceptions. Because the SF-36 has been applied globally and has validated normative values for several other diseases, it will enable us to place our findings about PASC in the broader context of relative burden of disease. Moreover, because the SF36 can be interpreted as a measure of health-related quality of life, it will enable comparisons of the cost and effectiveness of our comprehensive rehabilitation intervention to those of other interventions for COVID and other diseases.

Secondary performance-based outcomes will include the **2-min step test**, the **5x sit-to-stand**, the **FVC/FEV1** assessment of pulmonary function;^40–43^ the **Trail Making Test**,^44^ a neuropsychological test that provides information about visual search speed, scanning, speed of processing, and mental flexibility, and the **Stroop Test**^45^ to assess executive functioning.

Other secondary patient reported outcomes include: the 0–100 visual analog scale for **pain** and pain interference;^46^ the modified **Borg Dyspnea** scale^47^ for shortness of breath upon exertion; the Montreal Cognitive Assessment-Blind (**MoCA Blind**),^48–49^ a 12-item assessment of global cognitive performance designed to detect mild cognitive dysfunction, the **Multifactorial Memory Questionnaire (MMQ**),^50^ a measure designed to assess subjective memory skills; the the Hospital Anxiety and Depression Scale (**HADS**),^51^, the short form of the DePaul Questionnaire to identify post exertional malaise (**DPEMQ)**,^52^ and the Fatigue Assessment Scale (**FAS**),^53^ a 10-item scale, to evaluate symptoms of chronic fatigue. Finally, the subscale on global impact on daily life of the Symptom Burden Questionnaire for Long COVID **(SBQ-LC**)^54^ and the **PROMIS self-efficacy** scale will be the source of information on symptom impact and their management.^55^ (Data on outcomes will be collected for all study participants at each of the three assessments.) Information to be obtained, by source and periodicity, is summarized in Table 2.

### Sample Size and Power Analysis

This study will apply strict RCT principles including randomization, careful tracking of all participants, and intent-to-treat analyses to ensure robust and unbiased results. The central study design is a difference-in-difference comparison of walking speed (6MWT) and general health & functioning (MOS-SF-36) with two main comparisons: (i) the pre- and post- treatment effects among subjects, and (ii) difference between those in the comprehensive rehabilitation and usual care arms over the 8-week and 90-day post-study entry period. To determine sample sizes, we specified a significance level of alpha = 0.025 for each test to account for multiple comparisons via the Bonferroni correction, two-tailed comparisons, within-participant correlation no larger than r = 0.5 between the two post-study entry evaluation time points, and auto-regressive (AR(1)) covariance structure. Various effect and sample sizes were considered based on differences from pre-post therapy values of the 6MWT from our pilot work. The proposed sample size (after anticipated attrition) of 120 treatment and 60 usual care/controls achieves 95% power to detect a 15% improvement in the 6MWT. For general health and functioning, the proposed sample will achieve 86% power for a standardized effect size of 0.3 SD (i.e., Cohen’s d effect size measured in units of standard deviation, SD) for pre-post comparisons and 0.2 SD for difference-in-difference in SF36 scores across subjects and usual care controls. Regression-adjusted effects are likely to show improved efficiency.

### Analytic Plan

The key dependent variables in the proposed RCT are the primary and secondary outcome measures described above evaluated at 8-weeks and 90 days post-study entry. Although the primary time point for treatment comparison is at 8-weeks, shortly after the end of the 6-week supervised rehabilitation intervention period, we will examine outcomes longitudinally to identify temporal or sustainability effects. Participants will be described according to relevant attributes. Further, simple comparisons of outcomes across intervention and usual care groups using intention-to-treat (ITT) principles will be provided. When conducting our ITT analyses, we will first identify baseline individual characteristics that differ between the two study groups using chi-square or t-tests where appropriate. Characteristics shown to be significantly different at a p < 0.20 level will be subsequently included in multivariate models.

#### Multivariable Analyses.

To determine differences between the 2 treatment arms analyses will be conducted separately at each follow-up period as well as longitudinally by pooling data from the two follow-up interviews. When estimating pooled models across different follow-up periods, attention will be paid to appropriately account for the effect of unobserved patient-specific time invariant effects. The Generalized Estimating Equations (GEE) method will be used, as appropriate, to account for such effects as well as clustering (i.e., multiple observations for individuals over time). The Hausman test will be applied to determine whether the fixed or the random effect model is the most appropriate for the data. Assuming the null hypothesis of orthogonality is maintained, GLS, GEE, or random effects logit models will be used in the estimation. Ordinary least squares will be applied to continuous dependent variables (e.g., SF-36 scores, overall and sub-components, pain VAS). For binary outcome measures, such as rank in the lowest quartile of specific scales (e.g., HADS), logistic regression models will be developed. All analyses will control for any patient-level variables found to differ by chance across intervention and usual care groups as well as pre- intervention level of the dependent variable. In addition, to examine whether intervention effects vary with the patient’s characteristics, we will re-estimate our models including interaction terms between treatment indicators and selected regressors (e.g., race/ethnicity, number of comorbidities). These additional regression analyses will provide important information on possible sub-group effects.

We hypothesize that the supervised rehabilitation intervention will yield effects in the direction of improved patients’ clinical performance and health & functioning (primary outcomes) as well as improved endurance, vitality, and cognitive functioning relative to both their pre-intervention levels and to the active control group. We also anticipate that patients in the intervention group will experience reduced anxiety and depression, reduced mood disturbances and improved self-efficacy compared to patients in the usual care group. We anticipate that these effects will be substantial (> 15%) and long-lasting for all outcomes relative to active controls. Although we anticipate that outcomes will on average improve over time among patients in the control group, our key hypothesis is that the trend towards superior outcomes among intervention individuals will outweigh any temporal improvements in outcomes among active control subjects.

## DISCUSSION

This research holds clear and timely clinical and policy relevance. As evidenced by the recent surges, it is unlikely that the release of COVID-19 vaccines lead to the end of PASC given the high numbers of individuals with continued symptoms several months from their initial infection. High rates of hesitancy and refusal to become vaccinated, both in the US and worldwide, and the relatively limited lasting effectiveness of existing vaccines to address emerging variants will also contribute to the persistence of PASC. At present, there is a dearth of rigorous scientific evidence about effective assessment and treatment of PASC that prevents the creation of evidence-based clinical guidelines. Despite their recent growth, outpatient COVID recovery programs remain an unproven proposition, so evaluating evidence of their impact would make a significant contribution to arguments for (or against) their broader diffusion, standardization of core practices, and reimbursement. Findings from our study will, rigorously and timely, fill this critical gap.

A major limitation of previous studies of is the lack of precise definitions of the interventions and the heterogenous outcomes across different studies. This investigation for the first time utilizes precisely defined interventions that are standard physical therapy and speech language pathology clinical programs. These can be widely adapted if they are found to be successful in ameliorating PASC symptoms. The outcomes used in this study are well validated and represent the areas of most impact from PASC. The attention of this study to incorporation of women and minority individuals will enhance the generalizability of these findings.

If successful, our intervention can be reliably scaled and easily incorporated into clinical care. Demonstrating the comparative- and cost-effectiveness of this program may facilitate greater reimbursement by insurers for this program and the number of visits demonstrated to have positive benefits. We anticipate the active program will result in greater improvements with long lasting positive outcomes. These findings can be used by health advocates, clinicians, hospitals, and health systems to advocate for evidence-based programs—even if more costly initially—for these persons suffering the long term sequalae of COVID infections.

## Figures and Tables

**Table 1.** Schedule of Enrollments, Assessments, and Interventions

	Enrollment	Allocation	Post Allocation	8 Week Follow-Up	90 Day Post-Intervention Follow-Up
**Timepoint:**	−t_1_	0	t_1–12_	t_13_	t_14_
Eligibility Screen	x	x			
Informed Consent		x			
Allocation of Intervention		x			
**Intervention:**					
WHO Self-Guided Program (Intervention A):					
In-person Therapy Intervention (Intervention B):			x		
**Outcome Measures:**					
6 Minute Walk Test		x		x	
5x Sit to Stand Test		x		x	
2 Minute Step Test		x		x	
Hand Grip Strength		x		x	
Quadricep Strength		x		x	
Forced Expiratory Volume (FEV1)		x		x	
Forced Vital Capacity (FVC)		x		x	
Stroop Effect Test		x		x	
Trail Making Test		x		x	
MOCA Blind		x		x	
BORG Dyspnea Scale		x		x	x
DePaul Symptom Questionnaire		x		x	
Symptom Burden Questionnaire for Long COVID (SBQLC): Impact on Daily Life Subscale		x		x	x
Multifactorial Memory Questionnaire (MMQ)		x		x	
Attention Questionnaire (APT II)		x		x	x
Hospital Anxiety and Depression Scale (HADS)		x		x	
Visual Analog Scale (VAS)		x		x	
Fatigue Assessment Scale (FAS)		x		x	
Patient Reported Outcome Measurement Information System (PROMIS): General Self-Efficacy Scale		x		x	
36 Item Short-Form Survey (SF-36)		x		x	x

**Table 2:** Information to be Obtained by Source and Periodicity

PATIENT BASELINE CHARACTERISTICS		
Demographics	Age, race/ethnicity, education, marital status; number of household members	PMBB & Baseline Patient Surveys
Socioeconomic	Health insurance; ecological measure of poverty (ADI); pre-COVID major usual activity pre-existing conditions, body mass index	
Health	Pre-existing conditions, body mass index	Baseline (pre-randomization) Patient Survey/Assessment
Risk Behaviors	Risk Behaviors: smoking, physical activity, alcohol use	
**OUTCOMES**		
Primary	*Performance-based*: Self-selected walking speed (**6MWT**)	Baseline (pre-randomization) Patient Survey/Assessment.
	*Patient-reported*: Health & Functioning (**MOS SF-36**)	
Secondary	*Performance-based*	Follow-up Surveys: 8-weeks & 90 days (post-study entry)
	**FVC/FEV1** pulmonary test	
	2-minute step test (2MST); 5x sit-to-stand	
	Trail Making; Stroop test	
	*Patient-reported*	
	Fatigue Assessment Scale (**FAS**)	
	De Paul Questionnaire - post-exertional malaise (**DPEMQ**)	
	Cognition: Montreal Cognitive Assessment (**MoCA-Blind**)	
	Memory: Multifactorial Memory Questionnaire (**MMQ**)	
	Symptom burden for Long COVID, global impact on daily life (**SBQ-LC**)	
	Pain & Pain interference (**Visual Analog Scale**)	
	Dyspnea: **Borg Scale**	
	Anxiety and Depression: **HADS**	
	Symptom Management: **PROMIS Self-Efficacy Scale**	
**PROCESS MEASURES**		
	Number and type of therapy sessions attended.	Study logs for intervention fidelity evaluation
	(supervised intervention group only)	
